# Factors associated with insomnia and suicidal thoughts among outpatients, healthcare workers, and the general population in Taiwan during COVID-19 pandemic: a cross-sectional study

**DOI:** 10.1186/s12889-022-14557-z

**Published:** 2022-11-21

**Authors:** Yi-Hsuan Lin, Jung-Sheng Chen, Po-Ching Huang, Mei-Yun Lu, Carol Strong, Chung-Ying Lin, Mark D. Griffiths, Nai-Ying Ko

**Affiliations:** 1 Department of Nursing, College of Medicine, National Cheng Kung University, No.1, University Road, Tainan, 701401 Taiwan; 2 The preparatory office of the NCKUH Geriatric Hospital, National Cheng Kung University Hospital, College of Medicine, National Cheng Kung University, No.138, Sheng Li Road, Tainan, Taiwan; 3 Department of Medical Research, E-Da Hospital, No.1, Yida Road, Kaohsiung, Taiwan; 4 Institute of Allied Health Sciences, College of Medicine, National Cheng Kung University, No.1, University Road, Tainan, 701401 Taiwan; 5 Department of Public Health, College of Medicine, National Cheng Kung University, No.1, University Road, Tainan, Taiwan; 6 Center for Infection Control, National Cheng Kung University Hospital, No. 138, Sheng Li Road, Tainan, 704302 Taiwan; 7 Biostatistics Consulting Center, National Cheng Kung University Hospital, College of Medicine, National Cheng Kung University, No. 138, Sheng Li Road, Tainan, 704302 Taiwan; 8 Department of Occupational Therapy, College of Medicine, National Cheng Kung University, No.1, University Road, Tainan, Taiwan; 9 International Gaming Research Unit, Psychology Department, Nottingham Trent University, 50 Shakespeare Street, Nottingham, UK

**Keywords:** Sleep disorder, Suicide, COVID-19, Mental health, Taiwan, Frontline worker

## Abstract

**Background:**

Insomnia and suicidal thoughts are two of the negative impacts that have been caused by the COVID-19 pandemic. Identifying the factors that contribute to these psychological problems may help develop strategies to sustain the mental health of the public. The present study examined the psychosocial impacts caused by the COVID-19 pandemic among different populations in Taiwan, and investigated the relationships between these psychosocial variables, insomnia, and suicidal thoughts.

**Methods:**

Between September 2020 and May 2021, online questionnaires including psychometrically validated scales were distributed to a convenience sample of outpatients (*n* = 205), healthcare workers (HCWs) (*n* = 500), and individuals in the general population (*n* = 1200) in Taiwan to collect data regarding their insomnia severity, suicidal thoughts, fear of COVID-19, trust of information, and resilience. Multivariate logistic regression methods were used to identify variables associated with suicidal thoughts and insomnia.

**Results:**

Greater fear of COVID-19 was significantly associated with suicidal thoughts: odds ratios (ORs) with 95% confidence interval (CI) = 1.155 (1.002–1.330) for outpatients; 1.127 (1.035–1.228) for HCWs; and 1.100 (1.130–1.222) for those in the general population. Higher resilience was significantly associated with lower insomnia: OR (95% CI) = 0.819 (0.725–0.926) for outpatients; 0.803 (0.728–0.887), for HCWs; 0.829 (0.785–0.875), and for those in the general population. In addition, there was a statistically significant association between insomnia diagnosis and greater fear of COVID-19 among HCWs (OR [95% CI] = 1.102 [1.062–1.144]) and those in the general population (OR [95% CI] = 1.079 [1.053–1.106]). Among outpatients, there was a statistically significant association between suicidal thoughts and lower trust of information (OR [95% CI] = 0.794 [0.646–0.976]), while among those in the general population there was a statistically significant association between suicidal thoughts and higher insomnia severity (OR [95% CI] = 1.175 [1.13–1.222]). A statistically significant association was also found between insomnia diagnosis and higher suicidal thoughts among those in the general population (OR [95% CI] = 3.455 [2.338–5.106]).

**Conclusions:**

Trust of information, fear, and resilience were important factors for suppressing suicidal thoughts and insomnia among the three study populations. Health policies that monitor psychological status and build resiliency of the public are recommended to help develop tailored strategies for different populations affected by the COVID-19 pandemic.

## Introduction

During the COVID-19 pandemic, uncertainty about the disease, strict enforcement of public health measures, and information overload may create psychosocial problems such as anxiety, fear and/or sleep problems [1–6]. Understanding the psychological consequences that accompany this global health crisis is critical to improve public well-being [7]. The impact of the pandemic on mental health has been highlighted and the building of a resilient health system in response to the pandemic is recommended [8].

Sleep has been an important issue during the COVID-19 outbreak: higher worries about COVID-19 led to higher severity of insomnia [9]. Also, psychological distress (e.g., fear, anxiety, and depression) were reported to be associated with sleep problems during the COVID-19 pandemic [10, 11]. Sleep problems during the COVID-19 pandemic had no gender differences [12], but the prevalence of sleep problems differed across COVID-19 patients (pooled prevalence 74.8%), healthcare workers (HCWs) (36%), and the general population (32.3%) [13].

Suicide has been another important public health concern during the COVID-19 pandemic. A systematic review suggested that the prevalence of COVID-19-related suicidal ideation was higher among those in the general population during the COVID-19 pandemic than before it [14]. Empirical research has indicated that the causes of suicide associated with the COVID-19 pandemic include the (i) negative socioeconomic impact of COVID-19, (ii) fear of getting infected, (iii) stress of unemployment, and (iv) emotional isolation caused by home confinement orders [15, 16].

Suicide and insomnia are significant mental health concerns which widely affect individual lives. However, during a public health crisis like the COVID-19 pandemic, these mental health concerns are likely to have been exacerbated. Fear of contracting the virus may amplify the psychological consequences of COVID-19 due to uncertain prognosis and stigma toward COVID-19 patients [14, 17]. Although communicating information about COVID-19 with the public is essential, information overload may lead to increased fear and increased suicide rates [18, 19]. Today’s media technologies enable individuals to receive information quickly from a variety of sources, and there is an important need for trustworthy health information during the pandemic. Therefore, the trustworthiness of information sources (i.e., those that can be considered the most honest and reliable such as that from reputable health organizations such as the World Health Organization, government health ministries, etc.) can be a significant factor in reducing public stress caused by COVID-19 [19].

Many individuals have lost their jobs, homes and/or loved ones during the pandemic, and their resilience level may make them adapt and heal differently [20]. From two major schools of thought regarding the concept of resilience [21], some define resilience as the human capacity to overcome negative events [22]; some define resilience as the process of coping with adversity and promoting well-being [23]. Nevertheless, during COVID-19 outbreak periods, resilience may protect individuals against psychological distress [15, 24].

Mental health impacts of COVID-19 were common across different populations (e.g., HCWs and university students), and the severity of mental health problems can vary depending on medical condition and risk of exposure to the virus [25, 26]. Studies had investigated psychosocial effects of COVID-19 on the general population, HCWs [2, 3, 10, 11, 13, 27–31], and COVID-19 patients [10, 13, 32]. These studies assessed mental condition such as anxiety, depression, and sleep problem caused by COVID-19 and/or the pandemic. Additionally, it has been suggested that these mental health problems caused by COVID-19 may co-occur. Moreover, these studies reported that the prevalence rates for (i) anxiety were 9.5–73.3% (HCWs) and 47% (COVID-19 patients); (ii) depression were 12.5 to 71.9% (HCWs) and 45% (COVID-19 patients); and (iii) sleep problems were 18–32.3% (general population), 31–36% (HCWs), and 34–74.8% (COVID-19 patients). Outpatients may also be vulnerable to mental health issues because of their pre-existing health conditions and higher risk of contracting the virus while attending hospital appointments during the pandemic [30, 33, 34]. However, to the best of the present authors’ knowledge, outpatients have remained an understudied population. In this context, a cross-sectional study on three populations was conducted to help develop tailored strategies for different populations during the COVID-19 pandemic.

The present study examined fear of COVID-19, trust in information, and resilience among three different Taiwanese populations (outpatients, general population, and HCWs). The relationships between the aforementioned psychosocial variables, insomnia, and suicidal thoughts were also investigated.

## Methods

### Participants and procedure

A cross-sectional study using online surveys was conducted to investigate the psychosocial effects of COVID-19 among three populations, including National Cheng Kung University Hospital (NCKUH) outpatients, NCKUH HCWs, and individuals in the general population.

HCWs and outpatients who had their email address registered in the NCKUH information system were recruited using convenience sampling. In addition, NCKUH outpatients had to have visited NCKUH hospital outpatient department at least once during the survey period. NCKUH HCWs had to have worked in the NCKUH during the survey period. Individuals in the general population were recruited using stratified sampling based on age and resident city to be a representative Taiwanese population. The sole inclusion criterion for the three populations was that the participants had to be aged 18 years or above. The online survey was conducted between September 2020 and May 2021, a relatively safe period during the COVID-19 pandemic in Taiwan [35]. The sample comprised a total of 1905 participants (205 outpatients; 500 HCWs; and 1200 individuals from the general population). Since NCKUH is a medical center, most of the HCWs in the study population had a high level of education due to workplace requirements. Moreover, the HCWs in the study consisted primarily of nurses and doctors working on the frontline.

### Measures

*Demographics:* Participants’ age, gender, and education level were collected using a demographic information sheet.

*Insomnia:* Insomnia severity was assessed using the Insomnia Severity Index (ISI) [27]. The ISI comprises seven items that evaluate insomnia. Individuals rate the items (e.g., *“How satisfied/dissatisfied are you with your current sleep pattern?”*) on a five-point scale from 0 (*no problem*) to 4 (*very severe problem*). The sum of the ISI scores ranges from 0 to 28, with a higher score indicating greater severity of insomnia [36]. A summed ISI score of 8 or above is interpreted as having insomnia. Therefore, a dichotomous score for ISI was created to compare individuals without insomnia diagnosis (ISI score of 7 and below) to those with insomnia diagnosis (ISI score of 8 and above) [37]. The internal consistency of the ISI in the present study was very good (Cronbach’s alpha (α) = 0.905 [outpatients]; 0.885 [HCWs]; and 0.894 [general population]).

*Suicidal thoughts:* Suicidal thoughts were assessed using one item (i.e., *“In the past week, have you had suicidal thoughts because of COVID-19?”*) rated on a five-point scale`; 1 (*not at all*), 2 (*slight*), 3 (*moderate*), 4 (*severe*), and 5 (*very severe*) [38]. A higher score indicates a higher severity of suicidal thoughts. Due to the highly skewed nature of the original five-point scale, a dichotomous score of suicidal thoughts was created to compare individuals with no suicidal thoughts to those with slight to very severe suicidal thoughts.

*Resilience:* Resilience was assessed using a self-developed four-item scale [39] with items rated on a five-point scale from 1 (*obviously worse*) to 5 (*obviously better*). More specifically, trait resilience [40, 41] was evaluated in the present study. The four items were *“How do you think your physical health compared to others before the COVID-19 pandemic?”*; *“How do you think your mental health compared to others before the COVID-19 pandemic?”*; *“How do you think your physical health compared to others in the last week?”*; and *“How do you think your mental health compared to others in the last week?”.* Items are summed to create a total score ranging from 4 to 20. Higher scores indicate a more resilient response to stressful events. The internal consistency of the four resilience items in the present study was good (α = 0.869).

*Trust in information:* Participants’ trust in information was assessed using the Believing COVID-19 Information Scale (BCIS) [42] comprising six different media sources. Items (e.g., *“How much do you believe in the COVID-19 information on television?”*) are rated on a five-point scale from 1 (*strongly disbelieve*) to 5 (*strongly believe*) and summed to create a total trust in information score. Higher scores indicate greater trust in information. The internal consistency of the six items on in the present study was good (α = 0.838).

*Fear of COVID-19:* Fear of COVID was assessed using the seven-item Fear of COVID-19 Scale (FCV-19S) [43]. The FCV-19S has been widely used in many countries and its psychometric properties are good in over 20 different languages including Chinese [44]. Items (e.g., *“I am afraid of losing my life because of COVID-19”*) are rated on a five-point scale from 1 (*strongly disagree*) to 5 (*strongly agree*). Higher scores indicate greater fear of COVID-19. The internal consistency of the FCV-19S in the present study was excellent (α = 0.904).

### Data analysis

The descriptive statistics of the socio-demographic information and scale scores (i.e., resilience, trust in information, fear of COVID-19, suicidal thoughts, and insomnia) of the three populations were calculated. Analyses of variance and χ^2^ tests with Bonferroni corrections were used to compare the differences between variables among the three populations. For each population, Pearson’s correlations were used to examine the linear relationships between the studied variables. Then, multiple linear regression models were constructed for each population to identify the variables associated with suicidal thoughts after adjusting for age, gender, and education level. Lastly, multivariate logistic regression models were constructed for each population to examine how the studied variables were associated with insomnia after adjusting for age, gender, and education level. All the statistics were carried out using SPSS Statistics 17.0.

## Results

### Demographic characteristics of study participants

Among the 205 NCKUH outpatients (mean age 34.82 years [SD = 10.12]), 34.6% were males and 84.9% had a college or higher education level. Among the 500 NCKUH HCWs (mean age 32.96 years [SD = 7.99]), 40% were males and 96.6% had a college or higher education level. Among the 1200 general population participants (mean age 39.56 years [SD = 9.58]), 47.5% were males and 75.4% had a college or higher education level. Significant differences among the three populations were found in their age, gender distribution, and educational level (*p* < 0.001). There were no statistical differences between the three populations in terms of trust in information and insomnia severity. The prevalence rates of insomnia were 39.5% among outpatients, 44.6% among HCWs, and 39.8% among those in the general population. However, the general population had the highest scores in resilience (*p =* 0.005), fear of COVID-19 (*p <* 0.001), and suicidal thoughts severity (*p =* 0.007) compared to HCWs and outpatients. Table 1 lists the detailed information of the three populations. Moreover, scores of resilience, trust of information, fear of COVID-19, and ISI were normally distributed (skewness = − 0.68 to 0.90 in outpatients; − 0.76 to 0.55 in HCWs; and − 0.44 to 0.91 in general population) (Table 2).

**Table 1:** Demographic information in the three populations

	1. Outpatients (***N*** = 250)	2. Healthcare workers^a^ (***N*** = 500)	3. General population (***N*** = 1200)	F or χ^**2**^ (***p***-value)	Post-hoc comparison^b^
	***Mean ± SD or n(%)***	***Mean ± SD or n(%)***	***Mean ± SD or n(%)***		
**Age**	34.82 ± 10.12	32.96 ± 7.99	39.56 ± 9.58	F = 97.89 (< 0.001)	3 > 1 > 2
**Gender**				χ2 = 238.71 (< 0.001)	
Male	71 (34.6)	40 (8)	570 (47.5)		3 > 1 > 2
Female	134 (65.4)	458 (92)	630 (52.5)		
**Education**				χ^2^ = 107.55 (< 0.001)	
Senior high or below	31 (15.1)	17 (3.4)	295 (24.6)		3 > 1 > 2
College or above	174 (84.9)	479 (96.6)	905 (75.4)		
**Resilience**	11.60 ± 2.79	12.01 ± 2.25	12.22 ± 2.65	F = 5.36 (0.005)	3 > 1
**Trust of information source score**	19.50 ± 3.79	19.54 ± 3.35	19.81 ± 3.35	F = 1.58 (0.207)	–
**Fear of COVID-19**	18.03 ± 6.77	17.98 ± 5.77	19.69 ± 5.72	F = 18.47 (< 0.001)	3 > 1; 3 > 2
**Suicidal thoughts severity**	1.07 ± 0.38	1.08 ± 0.36	1.15 ± 0.54	F = 5.00 (0.007)	3 > 2
**Insomnia Severity Index score**	6.86 ± 5.19	7.15 ± 4.64	6.84 ± 5.3	F = 0.66 (0.518)	–
**Insomnia prevalence**^c^	81 (39.5)	223 (44.6)	477 (39.8)	χ^2^ = 3.64 (0.16)	–

^a^With missing value of age (*n* = 3), gender (*n* = 2), and education (*n* = 4)

^b^With Bonferroni correction

^c^With cut-off point = 8

**Table 2:** Descriptive statistics of the scale variables

	Min	Max	Mean	SD	Skewness	Kurtosis
**Outpatients**						
**Resilience score**	4	20	11.60	2.79	0.21	1.17
**Trust of information**	6	29	19.50	3.79	− 0.68	1.63
**Fear of COVID-19 score**	7	35	18.03	6.77	0.14	− 0.68
**Suicide score**^a^	1	5	1.07	0.40	6.88	55.34
**ISI score**	0	26	6.86	5.19	0.90	0.53
**Healthcare workers**						
**Resilience score**	4	20	12.01	2.25	0.48	2.40
**Trust of information**	6	30	19.54	3.35	− 0.76	1.58
**Fear of COVID-19 score**	7	35	17.98	5.77	0.06	−0.29
**Suicide score**^a^	1	3	1.08	0.36	4.80	22.23
**ISI score**	0	23	7.15	4.64	0.55	− 0.10
**General population**						
**Resilience score**	4	20	12.22	2.65	0.37	1.68
**Trust of information**	6	30	19.81	3.35	−0.44	2.20
**Fear of COVID-19 score**	7	35	19.69	5.72	0.15	0.13
**Suicide score**^a^	1	5	1.15	0.54	4.36	21.43
**ISI score**	0	28	6.84	5.30	0.91	0.55

^a^As continuous data. *ISI* Insomnia Severity Index

### Correlations between the study variables

Regarding the correlations between these studied variables, fear of COVID-19 was positively correlated with the trust in information (*r* = 0.332, *p* < 0.01, for outpatients; *r* = 0.118, *p* < 0.01, for HCWs; *r* = 0.17, *p* < 0.01, for general population) and insomnia severity (*r* = 0.166, *p* < 0.05, for outpatients; *r* = 0.342, *p* < 0.01, for HCWs; *r* = 0.231, *p* < 0.01, for general population). Resilience was negatively correlated with insomnia severity (*r* = − 0.342, *p* < 0.01, for outpatients; *r* = − 0.325, *p* < 0.01, for HCWs; *r* = − 0.295, *p* < 0.01, for general population). Moreover, among outpatients, resilience was negatively correlated with suicidal thoughts severity (*r* = − 0.169, *p* < 0.05) and trust in information (*r* = − 0.181, *p* < 0.01). Among HCWs, suicidal thoughts severity were positively correlated with fear (*r* = 0.139, *p* < 0.01) and insomnia severity (*r* = 0.108, *p* < 0.05); resilience was negatively correlated with fear (*r* = − 0.187, *p* < 0.01). Among those in the general population, all the correlations were significant except for the relationship between resilience and fear of COVID-19 (Table 3).

**Table 3:** Correlation matrix of covariates and studied variables for the three populations

	1^a^	2^b^	3^b^	4^a^	5^a^	6^a^	7^a^	8^a^
**Outpatients**								
**1. Age**	–							
**2. Gender**	−0.056	–						
**3. Education**	−0.196**	−0.103	–					
**4. Resilience**	0.176*	−0.125	0.098	–				
**5. Trust of information**	0.032	0.075	0.062	0.128	–			
**6. Fear of COVID-19**	−0.091	0.132	0.051	−0.026	0.332**	–		
**7. Suicidal thoughts severity**	−0.096	−0.091	− 0.034	−0.169*	− 0.181**	0.032	–	
**8. Insomnia severity**	−0.06	0.034	0.022	−0.342**	0.026	0.166*	0.108	–
**Healthcare workers**								
**1. Age**	–							
**2. Gender**	−0.014	–						
**3. Education**	0.107*	−0.076	–					
**4. Resilience**	0.002	−0.08	0.086	–				
**5. Trust of information**	−0.021	0.108*	−0.068	0.006	–			
**6. Fear of COVID-19**	0.05	0.158**	−0.149**	−0.187**	0.118**	–		
**7. Suicidal thoughts severity**	−0.055	−0.036	− 0.085	−0.026	− 0.066	0.139**	–	
**8. Insomnia severity**	0.067	0.053	−0.153**	−0.325**	− 0.023	0.342**	0.108*	–
**General population**								
**1. Age**	–							
**2. Gender**	−0.134**	**–**						
**3. Education**	−0.114**	−0.049	–					
**4. Resilience**	0.063*	−0.108**	0.068*	–				
**5. Trust of information**	−0.005	− 0.008	0.023	0.157**	–			
**6. Fear of COVID-19**	−0.053	−0.019	− 0.064	−0.028	0.17**	–		
**7. Suicidal thoughts severity**	−0.036	−0.024	0.007	−0.156**	− 0.084**	0.191**	–	
**8. Insomnia severity**	−0.009	0.049	−0.013	−0.295**	− 0.059*	0.231**	0.322**	–

* *p* < 0.05

**p < 0.01

^a^With Pearson correlation

^b^With Spearman correlation

### The associations between suicidal thoughts with other variables

Table 4 shows the variables that were associated with suicidal thoughts among the three populations after adjusting for age, gender, and education level. The results of the logistic regression showed that higher fear of COVID-19 (outpatient: odds ratio [OR] = 1.155, 95% confidence interval [CI] = 1.002–1.33; HCWs: OR = 1.127, 95% CI = 1.035–1.228; general population: OR = 1.1, 95% CI = 1.13–1.222) was significantly associated with suicidal thoughts among the three populations after accounting for trust of information, resilience, and insomnia. In addition, lower trust of information (OR = 0.794, 95% CI = 0.646–0.976) was significantly associated with suicidal thoughts among outpatients, but not among the other two populations. Furthermore, while all studied variables were correlated with suicidal thoughts in the general population, only higher insomnia severity (OR = 1.175, 95% CI = 1.13–1.222) was significantly associated with suicidal thoughts when all the studied variables were considered.

**Table 4:** Logistic regression of variables associated with suicidal thoughts

	OR	95% CI	***p***-value
**Outpatients** [variance inflation factor (VIF) = 1.041–1.184, χ^2^ = 22.711 (*p* = 0.002)]			
**Age (years)**	0.942	0.855–1.038	0.226
**Gender**			
Male	0.172	0.032–0.943	0.043
Female	*Reference*		
**Education**			
Senior high or below	3.66	0.625–21.442	0.15
College or above	*Reference*		
**Resilience**	0.739	0.545–1.003	0.052
**Trust of information**	0.794	0.646–0.976	0.028
**Fear of COVID-19**	1.155	1.002–1.33	0.046
**Insomnia severity**	1.054	0.917–1.21	0.459
**Healthcare workers** [VIF = 1.033–1.25, χ^2^ = 19.313 (*p* = 0.007)]			
**Age (years)**	0.948	0.891–1.008	0.088
**Gender**			
Male	0.413	0.107–1.597	0.2
Female	*Reference*		
**Education**			
Senior high or below	3.373	0.657–17.306	0.145
College or above	*Reference*		
**Resilience**	1.004	0.816–1.237	0.968
**Trust of information**	0.91	0.813–1.018	0.098
**Fear of COVID-19**	1.127	1.035–1.228	0.006
**Insomnia severity**	1.062	0.965–1.169	0.216
**General population** [VIF = 1.032–1.168, χ^2^ = 132.043 (*p* < 0.001)]			
**Age (years)**	0.978	0.955–1.003	0.084
**Gender**			
Male	0.673	0.43–1.052	0.082
Female	*Reference*		
**Education**			
Senior high or below	0.832	0.482–1.435	0.508
College or above	*Reference*		
**Resilience**	0.958	0.883–1.04	0.305
**Trust of information**	0.988	0.929–1.051	0.702
**Fear of COVID-19**	1.1	1.056–1.146	< 0.001
**Insomnia severity**	1.175	1.13–1.222	< 0.001

### The associations between insomnia with other variables

The logistic regression analyses showed that lower resilience (outpatient: odds ratio [OR] = 0.819, 95% confidence interval [CI] = 0.725–0.926; HCWs: OR = 0.803, 95% CI = 0.728–0.887; general population: OR = 0.829, 95% CI = 0.785–0.875) was significantly associated with insomnia diagnosis among the three populations. Moreover, higher fear of COVID-19 (HCWs: OR = 1.102, 95% CI = 1.062–1.144; general population: OR = 1.079, 95% CI = 1.053–1.106) was significantly associated with insomnia diagnosis among HCWs and the general population. In addition, higher suicidal thoughts were also significantly associated with insomnia diagnosis among the general population (OR = 3.455, 95% CI = 2.338–5.106) (Table 5).

**Table 5:** Logistic regression of variables associated with insomnia

	OR	95% CI	***p***-value
**Outpatients** [variance inflation factor (VIF) = 1.043–1.196, χ^2^ = 17.373 (*p* = 0.015)]			
**Age (years)**	1.011	0.979–1.043	0.518
**Gender**			
Male	1.284	0.684–2.409	0.436
Female	*Reference*		
**Education**			
Senior high or below	0.953	0.394–2.308	0.916
College or above	*Reference*		
**Resilience**	0.819	0.725–0.926	0.001
**Trust of information**	0.978	0.898–1.064	0.601
**Fear of COVID-19**	1.033	0.985–1.084	0.181
**Suicidal thoughts severity**	1.562	0.649–3.759	0.320
**Healthcare workers** [VIF = 1.034–1.117, χ^2^ = 72.273 (*p* < 0.001)]			
**Age (years)**	0.998	0.974–1.023	0.891
**Gender**			
Male	0.809	0.381–1.719	0.582
Female	*Reference*		
**Education**			
Senior high or below	1.198	0.423–3.394	0.734
College or above	*Reference*		
**Resilience**	0.803	0.728–0.887	< 0.001
**Trust of information**	0.981	0.925–1.042	0.535
**Fear of COVID-19**	1.102	1.062–1.144	< 0.001
**Suicidal thoughts severity**	2.024	1.067–3.838	0.031
**General population** [VIF = 1.032–1.095, χ^2^ = 183.248 (*p* < 0.001)]			
**Age (years)**	1.011	0.997–1.025	0.111
**Gender**			
Male	0.886	0.687–1.142	0.349
Female	*Reference*		
**Education**			
Senior high or below	0.862	0.639–1.163	0.331
College or above	*Reference*		
**Resilience**	0.829	0.785–0.875	< 0.001
**Trust of information**	1	0.961–1.042	0.982
**Fear of COVID-19**	1.079	1.053–1.106	< 0.001
**Suicidal thoughts severity**	3.455	2.338–5.106	< 0.001

## Discussion

This present study conducted an online survey to examine the relationship between fear of COVID-19, trust in information, and resilience with insomnia and suicidal thought among different populations in Taiwan, including hospital outpatients, HCWs, and those in the general population. While the general population had the highest resilience score of the three groups, the general population also had the highest scores for fear of COVID-19 and suicidal thoughts. This is perhaps understandable because resilience is not a fixed mental health status that maintains individuals in a good mood all the time, but keeps individuals going in the long run in the face of any adversity [24]. Findings from the present study suggested that higher fear of COVID-19 was associated with suicidal thoughts, and a lower level of resilience was associated with higher likelihood of experiencing insomnia symptoms. It is recommended that these constructs are targeted to develop tailored strategies for different populations.

The results of the present study indicated that higher fear of COVID-19 was significantly associated with suicidal thoughts among all three populations, which is consistent with findings from the previous studies [28, 45–47]. In addition, findings indicated that increased insomnia symptoms were significantly associated with suicidal thoughts among those in the general population, which is also in line with the previous studies [20, 28]. The negative association between trust in COVID-19 information and suicidal thoughts among outpatients may perhaps be explained by health education they received when visiting the hospital [48]. The psychological consequences of stress-inducing information about COVID-19 may be mitigated if the government adequately communicated and monitored the content of COVID-19 information [18].

According to the (Taiwanese) Ministry of Health and Welfare report, the suicide rate in Taiwan in 2020 did not increase as compared to the rate in the previous year, which may be interpreted as the COVID-19 pandemic having little impact on suicide rate. In addition, the results of a study conducted in Taiwan showed a slight decrease in the annual suicide rate following the COVID-19 outbreak, although the suicide rates increased for individuals in younger and older age groups [49]. Experts in the field of suicidology have recommended that public health officials should still implement mental health interventions to maintain psychosocial well-being and prevent suicide among vulnerable individuals and communities [17].

In relation to other variables, findings in the present study also indicated that lack of resilience was significantly associated with insomnia among all three studied populations. This finding is consistent with previous studies conducted online with no participation restrictions, with higher levels of resilience accompanied by fewer insomnia symptoms [20]. Moreover, higher fear of COVID-19 was also associated with insomnia among HCWs and those in the general population. Fear and anxiety about COVID-19 have been found to be significant associated with sleep problems among healthcare professionals working on the frontline in hospitals and health centers [27]. A systematic review and meta-analysis of studies focusing on the impact of fear of COVID-19 also reported that fear of COVID-19 was positively associated with insomnia, as well as depression, anxiety, and other mental health problems [11]. These results supported the findings of the present study.

Sleep problems accompany many other types of psychological distress [10, 11]. A recently published systematic review reported an interrelationship between insomnia and post-traumatic stress disorder (PTSD) [50]. Additionally, evidence from prospective studies has shown a bidirectional association between insomnia and other physical (e.g., headache, asthma) or mental (e.g., anxiety, depression) health problems [51, 52]. Consequently, monitoring the psychosocial needs of the general public is critical because insomnia is one of the psychological effects of COVID-19 that can last for a long time [7].

The prevalence of insomnia among the general population (39.8%) and HCWs (44.6%) in the present study were in line with a systematic review and meta-analysis of studies on sleep problems among different groups of individuals during the COVID-19 pandemic, which showed that the pooled prevalence of sleep problems was 37% for the general population, 43% for HCWs and 57% for COVID-19 patients [10]. The insomnia prevalence among outpatients (39.5%) was the lowest among the three populations in the present study. However, one study in Turkey reported that 48.6% of cancer outpatients had insomnia, and another study in Malaysia reported that 80.9% of depression outpatients had insomnia during the pandemic [34, 53]. Therefore, differences in the prevalence rates of insomnia may be due to differences in individuals’ underlying medical conditions [25, 26]. It is therefore possible that immunocompromised cancer outpatients and emotional vulnerable depression outpatients were more likely to be affected by COVID-19 pandemic in relation to their sleep.

Previous studies have shown that pandemic outbreaks such as severe acute respiratory syndrome (SARS) and the H1N1 pandemic can result in mental health problems among individuals [26]. Despite the unprecedented challenges posed by the COVID-19 pandemic, the Taiwanese government’s efficiency in implementing public health measure and the Taiwanese people’ compliance with regulations have mitigated the impact of the disease. Having experience with past pandemics, the public are likely to develop similar but hopefully better coping strategies.

### Strengths and limitations

The present study had some limitations. First, the cross-sectional design of this study was unable to establish causal relationships between the variables assessed. Second, there may be social desirability bias and recall bias because all the data were self-report and participants’ responses were not objectively verified. Third, the findings of the study cannot be generalized to the entire Taiwanese population because participants were of a younger age group. A possible explanation is that online surveys may prevent people who are older and unfamiliar with smartphones or computers from participating. Furthermore, given that this present study only recruited Taiwanese participants, the data may not be representative of the same three populations in other countries. Finally, suicidal thoughts were assessed using a single item rather than a psychometrically validated scale. However, results from past research suggest that if the construct being assessed is subjective and well understood, then a single item measure might be a reasonable substitute for a longer assessment [54]. For instance, one study showed that a single-item to assess depression was sufficient to assess the mood of patients with anxiety disorders [55].

Future studies are needed to understand more thoroughly the mediation mechanism underlying these psychosocial factors. For example, the present study carried out an exploratory examination as to whether insomnia severity mediated the relationship between fear of COVID-19 and suicidal ideation. The preliminary results indicated that in the relationship between fear of COVID-19 and suicidal ideation, insomnia severity was not a significant mediator for HCWs (95% bootstrapping of the indirect effects = − 0.008, 0.040) but was a significant mediator for those in the general population (95% bootstrapping of the indirect effects = 0.025, 0.047). However, the present study was a cross-sectional study design and was unable to provide evidence regarding causal relationship. Therefore, future studies using longitudinal design are needed to further examine the potential mediation mechanisms between fear of COVID-19, insomnia severity, and suicidal ideation.

The present study had some strengths because it compared three different Taiwanese populations (i.e., hospital outpatients, HCWs, and the general population during the same period of COVID-19 pandemic). This may offer insights into the psychological characteristics and prevention targets of different populations affected by the COVID-19 pandemic. Given that there were positive associations between fear of COVID-19 and suicidal ideation, and negative associations between resilience and insomnia for all three groups in this present study, early detection of the psychosocial needs of different populations requires attention, and strategies to improve individual and community resilience should be implemented.

## Conclusion

The present study examined associations between COVID-19-related psychosocial variables, suicidal thoughts, and insomnia among the general population, HCWs, and hospital outpatients in Taiwan. The findings suggested that higher levels of fear of COVID-19 were related to suicidal thoughts. Furthermore, the lower the resilience of the individual, the higher the chances of the individual experiencing insomnia symptoms. It is recommended that health policies monitoring mental health and that build resiliency among individuals are implemented, and to identify risk factors for each population group to develop tailored strategies in the context of COVID-19 pandemic.

## Data Availability

The data can be requested from the corresponding author with proper use.

## Abbreviations

COVID-19: Coronavirus disease of 2019
HCWs: Healthcare workers
ISI: Insomnia Severity Index
BCIS: Believing COVID-19 Information Scale
FCV-19S: Fear of COVID-19 Scale
OR: Odds ratio
CI: Confidence interval
